# Do Not Pass Up the Opportunity!

**DOI:** 10.1007/978-3-030-65355-2_3

**Published:** 2021-03-20

**Authors:** Maurice Adams

**Affiliations:** 1 Tilburg University, Tilburg, The Netherlands; 2 Tilburg University, Tilburg, The Netherlands; 3 Tilburg University, Tilburg, The Netherlands; 4 Tilburg University, Tilburg, The Netherlands; Department of Public Law & Governance, Tilburg Law School, Tilburg, The Netherlands

## Abstract

A much-cited article on the website “Inside Higher ED” outlines fifteen scenarios for higher education in the coming period (Malony and Kim, www.insidehighered.com/digital-learning/blogs/learning-innovation/15-fall-scenarios; 2020). The continuum ranges from education completely back to normal to education completely at a distance. Full return to normal is not on the horizon; full distance education is possible but not optimal for most educational programs; and somewhere in between is quite a challenge. However, it is striking that the continuum focuses entirely on official educational activities and that there is no attention for informal educational activities: a neglect that is reflected in the current discussions about the future of our education. This chapter is about this neglect and the proposition is that the coronavirus crisis offers significant opportunities for a “new educational common” at Tilburg University in this respect.

A much-cited article on the website “Inside Higher ED” outlines fifteen scenarios for higher education in the coming period (Malony and Kim 2020). The continuum ranges from education completely back to normal to education completely at a distance. Full return to normal is not on the horizon; full distance education is possible but not optimal for most educational programs; and somewhere in between is quite a challenge. However, it is striking that the continuum focuses entirely on official educational activities and that there is no attention for informal educational activities: a neglect that is reflected in the current discussions about the future of our education. This chapter is about this neglect and the proposition is that the coronavirus crisis offers significant opportunities for a “new educational common” at Tilburg University in this respect.

## The Socialization Function

The Dutch educationalist Gert Biesta argues that education is not just about the transfer of knowledge and about skills training. Socialization also plays a crucial role.^1^ Indeed, students should be prepared for their future professions through knowledge and skills. But they should also be initiated into values and traditions, enabling them to participate in existing and future social, cultural, and political structures.

The efforts of Dutch higher education institutions in recent months have focused mainly on knowledge transfer. Moreover, an enormous effort has been made to ensure that all exams were properly conducted. The prevention of study delays and the ambition to guarantee the quality of our diplomas played a crucial role in all of this. Now that the new academic year is approaching, it is important to think about the aforementioned socialization function of education. It is a theme that, until recently, was beyond the horizon of higher education: in the old common, we took socialization for granted all too easily. And to some extent it indeed was sort of easy: as (a) a self-evident part of regular educational processes and (b) a bycatch of a well-developed student life.

In a recent report of the Dutch Education Council—an advisory body for Dutch government—the “socialization challenge” is also addressed through this two-pronged approach (Onderwijsraad 2020). The Education Council emphasizes that although the coronavirus crisis brings students many new and unique life experiences, social distancing and distance learning are crippling the relationships and interactions that promote a profound educational experience. With reference to the above-mentioned Biesta, among others, the Council states that

…precisely by learning with each other, by interacting with each other and with their teachers/lecturers, and by being part of a group, students develop themselves. The fact that (…) young people now meet their peers a lot less often affects them in their cognitive and socio-emotional development and the development of their brains (Onderwijsraad 2020)*.*

While the Education Council, in the above-mentioned quote, mainly points out how the socialization aspects of education are shaped through the official route, it also reminds us that studying is about discussions in the coffee corner and about an active student life as well:

It is precisely the activities of student and study associations and other extra-curricular activities that contribute to social bonding, to deepening and broadening the content of their studies, to an international experience, to career and professional orientation, and to the formation of future professional networks (Onderwijsraad 2020)*.*

Here, the Council is referring to the university as a social meeting place, outside the curriculum. And hopefully across social dividing lines too: the coronavirus crisis indeed made abundantly clear that diversity and inclusion is still a huge challenge for the weaker groups in society (Aarts et al. 2020).

## Enhancing Our Educational Profile

Group formation and social bonding are thus established within and outside the official frameworks of education. As far as I am concerned, the current situation reveals just how much we have lost sight of the extracurricular aspects of education in the past. It is, therefore, also no coincidence that the extracurricular aspect of education in the public and academic debate on the future of our education is currently almost completely out of sight. In other words, and on a positive note, there is now an opportunity to take responsibility for stimulating and facilitating the more informal socialization aspects of education.

Such an ambition could fit nicely into TEP: the Tilburg Educational Profile (Tilburg University 2017), with which Tilburg University positions itself in the educational landscape. TEP stands for an educational vision in which knowledge, skills, and character are central. Whereas knowledge transfer and skills training are self-evident in an environment of higher education, the character aspect focuses on the development of the critical, self-aware attitude of our students towards the society in which they function (Understanding Society). It is about students who are committed and socially critical, who are able to work together in various professional settings (as citizens of this world as it were), and who are able to fit into existing structures as citizens.

However, TEP focuses primarily on what we do within the official and formal frameworks of education. My proposal is, therefore, to now develop TEP through a process of extracurricular activities; you could call it the living or sticky campus: a place where students want to spend time, even if they do not have formal education! Precisely such a living campus offers opportunities for creating an environment outside the official frameworks in which we, as a university, can live up to what it means to educate critically committed students.

Let me give some examples: it could be about better facilitating a culture of debate and discussion about the major societal challenges we are facing; it could be about better-facilitating networks in which students coach and support each other; we could develop stronger cooperation with regional or national cultural institutions (museums, music centers). Connecting and collaborating with the city of Tilburg and other Tilburg institutes of higher education should be a high priority too! It may also be about offering hands-on skills that make it possible for graduates to enter professional life effectively: language skills for all students (including Dutch language and culture for international students), digital literacy, and labor market orientation. We should also include alumni and career services in all of this. In addition, international students can be linked to teams of Dutch students for a cultural exchange and for help with planning and interaction with the university, et cetera. In any case, study and student associations must be intensively involved in all of this or even be in charge. When developing ideas it would be helpful to start mapping the existing activities via a matrix: on the one hand with respect to formal and informal, physical and digital activities, on the other hand in terms of study program, School, or university. From there, gaps can be identified, ideas developed, and structures build. In any event, because of its size and compactness, the Tilburg University campus offers unique opportunities to profile itself vis-a-vis other universities in this regard. This of course requires an investment, but character building can then really become a distinctive feature. I add that it could also result in students feeling more involved with their university, with ultimately fewer dropouts (Tinto 1993; Connolly 2016).

An important question is still lingering in the air: what to do when health risks might force us to continue functioning online in the foreseeable future? What if the campus is still a physically quiet place, like in the spring of 2020? As far as activities outside the curriculum are concerned, there is no reason why the examples I gave in a previous paragraph of this contribution cannot serve, mutatis mutandis, as inspiration here. Although suboptimal, most if not all of the examples can be realized through developing a virtual environment (Last 2020; Kappe 2020).

## Democracy and the Rule of Law

For those who now think that socialization is a sort of training in conformism^2^: that is not the point here. For me, the above relates to what constitutes the threshold of a society that is truly committed to democracy and the rule of law. Socialization is not about conformism or about ensuring that there are no tensions between people, cultures, groups, religions, or generations. On the contrary, a democracy unavoidably leads to vigorous discussion and debate and to the cacophony and false notes. It is a permanent exercise in trial and error. However, we must use these tensions positively and deal with them in a non-violent manner. Making that possible is at the heart of any democracy that is committed to the rule of law. Simply because only under that condition can we have a common future in a world that is inevitably divided. Seen in this way, socialization is a true learning experience. It is not just about the joys of reaching your aims and success in collaborating with others. It is also about experiencing that you are always dependent on others, that you cannot always get your way, and that you can be disappointed when working together, but that, in spite of these, you can still have a shared and worthwhile future.

To conclude: we have to invest in the logical next steps that will enable us to make a qualitative leap in education in the medium and long term. Socialization in the context of education, stimulated through formal but certainly also informal or extracurricular means, is an inseparable part of this. As the editors of this volume write in their introduction, the capacity to anticipate changes is of the utmost importance now (Aarts et al. 2020). Do not pass up the opportunity!

## Footnotes

1 Biesta (2006) also identifies subjectification as a main aim of education: this refers to emancipation and freedom and the responsibility attached.

2 The description by the above-mentioned Biesta might suggest this: after all, according to him students need to be initiated into values and traditions, enabling them to participate in existing social, cultural, and political structures. Onderwijsraad (2020). Vooruitzien voor jongere generaties, The Hague. See also website: www.onderwijsraad.nl/publicaties/adviezen/2020/06/09/advies-vooruitzien-voor-jonge-generaties
